# The Efficient Method for Simultaneous Monitoring of the Culturable as Well as Nonculturable Airborne Microorganisms

**DOI:** 10.1371/journal.pone.0082186

**Published:** 2013-12-20

**Authors:** Barbara Hubad, Aleš Lapanje

**Affiliations:** 1 Institute of Microbial Sciences and Technologies, Domžale, Slovenia; Harvard Medical School, United States of America

## Abstract

Cultivation-based microbiological methods are a gold standard for monitoring of airborne micro-organisms to determine the occupational exposure levels or transmission paths of a particular infectious agent. Some highly contagious microorganisms are not easily culturable but it is becoming evident that cultivation and molecular methods are complementary and in these cases highly relevant. We report a simple and efficient method for sampling and analyzing airborne bacteria with an impactor-type high-flow-rate portable air sampler, currently used for monitoring culturable bacteria or fungi. A method is reported for extraction of nucleic acids from impacted cells without prior cultivation and using agarose as a sampling matrix. The DNA extraction efficiency was determined in spiked samples and, samples taken from a wastewater treatment plant and an alpine area. The abundance, diversity and quantity of total bacteria were analysed by a quantitative polymerase chain reaction, and by construction and analysis of clone libraries. The method does not interfere with downstream PCR analysis and can cover the gap between traditional culture and molecular techniques of bioaerosol monitoring.

## Introduction

Effective monitoring of bioaerosols requires efficient collection of microorganisms and an appropriate technique for their analysis [1], [2]. There is no standard method for collecting bioaerosols, but culture dependent methods are generally recognized as the gold standard in monitoring clean rooms (e.g. pharmaceutical and medical instrumentation production facilities, operating rooms and hospital indoor air), since isolation and cultivation of a specific organism is currently the only validated approach to link causative agents to a particular disease. However, some bacteria, including pathogens such as *Legionella pneumophila*, can be in a “viable but nonculturable” physiological state and others, such as *Mycobacterium tuberculosis* are initially hard to cultivate. Although cultivation techniques can be used to isolate most of the microorganisms that are of concern to humans, a majority of bacteria, which arguably are the most environmentally relevant, cannot be cultivated at all [3]–[7]. This suggests the need to improve current methods for bioaerosol analysis. Introduction of molecular methods based on DNA isolated directly from environmental samples of culturable and non-culturable bacteria, is expected to provide more information than each one separately [1], [7].

Methods currently used to collect airborne bacteria include sampling with filters, liquid impingement, impaction on solid agar or passive sedimentation. However, when both culturable and non-culturable fractions of bacteria are desired, liquid impingement is most frequently used [7], [8]. The impingement samplers are less robust which results in several disadvantages such as rapid evaporation of sampling liquid, samplers are typically not battery driven and can be used only in vertical position. In these samplers the evaporation of sampling liquid limits sampling time and lowers collection efficiency. Moreover, additional handling of liquid, such as inoculation onto growth media, is needed. Impactor samplers can overcome these obstacles, but are currently used mainly for collection and analysis of airborne microorganisms, which can be grown on agar growth media [9], [10]. In favor of impactor based sampling method, diversity of culturable bacteria was reported to be higher then by air filtration method as well as by impingement [9]. Despite the advantages of impactors used for collection and characterization of culturable bacteria, only three studies have been published that extend their use in molecular approaches based merely on isolated DNA from collected airborne bacteria without prior cultivation [9], [11], [12]. In each case, solid gelatin or liquid mineral oil were used as an impactor matrix, which were chosen based on low melting point or low evaporation rate, respectively. Accordingly, mineral oil enables longer sampling times, but it cannot provide solid support during impaction. This results in uneven distribution of oil in impaction holders and liquid loss during handling of the sampling liquid, which presumably influences DNA extraction efficiency [12]. Gelatin however, has a solid structure at room temperature and low melting point (in a range of 30–37°C), which is beneficial for DNA extraction, since it simplifies dissolution of the solid matrix [13]. Accordingly, the solid matrix is the most preferable for sampling. However, according to our knowledge the poorly defined chemical characteristics of gelatin, which is composed of mixed size and differentially branched polymeric matrix, as well as inhibition of PCR due to high protein content, is especially pronounced in samples with low numbers of cells [14]. If needed to use cultivation in parallel to molecular methods, the low melting point of gelatin limits its use at temperatures of 37°C and above, which is especially problematic for incubation of pathogenic bacteria. Additionally, gelatin can be degraded by many bacteria especially eutrophic ones resulting in liquefied medium [15]. Therefore it is of certain need to use alternative matrix with very similar properties as gelatin but without drawbacks described above.

An ultimate approach to bioaerosol monitoring would be simultaneous analysis of air samples by classical culturing methods and by molecular methods without additional handling. One favorable approach would be to use an impactor sampler with classic growth media, from which in parallel one part of the sample would be used in cultivation approach and the other for direct DNA extraction. However, agar used to solidify nutrient media is not molecular biology grade product and as such is inappropriate to be used in DNA based analysis. Since this methodology is currently unavailable, we have sought to develop and evaluate a new method for monitoring total and culturable airborne bacteria, adapted to the portable impaction based air sampler. The method reported here is capable of speeding up the detection of the source and identification of a particular microorganism.

## Materials and Methods

### Preparation of agarose matrix

RCS High Flow plastic strips (impaction holders) (Biotest, Germany) were used for agarose impaction matrix. The impaction holders are comprised of two rows of 17 wells, each containing 250 µL of matrix. The impaction matrix was prepared with analytical grade low melting point (LMP) agarose (Promega) in DNase- and RNase-free 1×TAE buffer (Sigma-Aldrich) and was sterilized by autoclaving for 20 min at 121°C. According to our preliminary studies, 0.7% (w/v) of agarose matrix was the most appropriate concentration, which provided a stable solid gel structure that withstanded air velocity during sampling. This concentration of agarose also minimized the interference with downstream methods since the large amounts of agarose in DNA extracts can inhibit PCR [16], [17]. Autoclaved agarose was kept liquid by incubation at 60°C and then applied to the empty, sterile impaction holders. A total of 8 mL of agarose was applied to each holder and the holder was then closed with a sterile cover and the contents were left to solidify at room temperature.

### Extraction of DNA from agarose matrices spiked with bacterial cells

To determine the most efficient protocol for extraction of DNA from agarose matrices, a random amount of *Escherichia coli* (ATCC 15597) and *Staphylococcus aureus* (ATCC 9144) in a range between 10^7^ and 10^8^ CFU were used. Overnight cultures grown in Luria-Bertani medium (Sigma-Aldrich) were applied to solid sterile agarose matrices and left for 1 hour at 22°C to soak completely into the matrix. The efficiency and yield of DNA extraction was determined by comparison of the obtained total DNA mass extracted from spiked agarose matrices with the amount of DNA extracted directly from bacterial cultures by the SmartHelix® Complex samples Kit (Sekvenator Ltd., Slovenia) DNA extraction protocol.

### Development and optimisation of DNA extraction protocol

#### Dissolution of the agarose matrix

To extract DNA from the captured cells, it is (i) necessary to collect cells from the matrix, (ii) to efficiently lyse the cells and (iii) to extract, purify and concentrate the DNA. The most appropriate way to obtain bacterial cells from the matrix is to completely dissolve and then degrade the agarose into its monomeric units. However, in our procedure, the amount of agarose that had to be dissolved exceeded the volumes typically used (0.5 mL) for direct DNA extraction. Therefore, adjustment of the reagent volumes and times for incubation at elevated temperatures to completely melt the agarose had to be determined from sample to sample. 0.7% LMP agarose with spiked bacterial cells was transferred from impaction holders into DNase- and RNase-free 10 mL tubes (Sarstedt). Acid and enzymatic hydrolysis of agarose was tested in combination with different temperatures to dissolve the agarose completely and at the same time to minimize prolonged exposure of sampled cells to elevated temperatures. Accordingly, for acid hydrolysis, 1 mL of 5.5 M guanidinium thiocyanate (GuSCN, pH 7) (Sigma-Aldrich) per 1 mL of agarose was added and then incubated at 65°C for 10 min, 15 min or 30 min. For enzymatic hydrolysis, agarose was first incubated at 65°C for 30 min or at 100°C for 1 min, 1.5 min or 5 min to dissolve and then left for 10 min to cool to 40°C. 7 U of β-agarase (Fermentas-Thermo Scientific) per 1 mL of dissolved agarose was then added and the mixture was incubated for 1 h or 1.5 h at 42°C.

#### Concentrating bacterial cells from dissolved agarose

The initial volume of agarose matrix expands to more than 8 times the volume that is normally used in most commercially available DNA extraction kits, and it was necessary to concentrate bacterial cells and cell debris by filtration prior to the DNA extraction. Accordingly, dissolved agarose samples from bacterial spiking experiments were passed through PES membrane filters (25 mm, pores 0.22 µm, Milipore). DNA was extracted from the filters by SmartHelix® Complex samples Kit (Sekvenator Ltd., Slovenia), since PES is completely dissolved in phenol/chloroform/isoamyl alcohol, the solvent in this DNA extraction kit.

#### Concentrating DNA in filtrates

Since microbial cells collected on the agarose matrix were exposed to elevated temperatures during the agarose melting, it was expected that a fraction of cells will have been lysed. Thus, DNA released from lysed cells freely passes through the 0.22 µm pore size PES filters during the cell concentration procedure and is found in the filtrate. Concentration of the DNA in the filtrate was achieved by centrifugation of samples in ultrafiltration columns (10,000 Mw cut-off, Vivaspin) at 9000 rpm for variable times, from 20 min to 60 min, until 100 µL of final DNA concentrate was obtained. The DNA concentration was determined in all samples by Quant-iT™ High-Sensitivity DNA Assay Kit Assay Kit using QubitTM fluorometer (Invitrogen) and the total extracted DNA mass was calculated.

#### Statistical analysis

Linear regression method was used to model the total DNA mass extracted from spiked agarose matrices as a function of total DNA mass extracted directly from bacterial cells. The suitability of linear regression line was evaluated based on random distribution of residual errors around the fitted values, normal distribution of residuals errors and absence of laverage points and outliers. The significance of correlation of was tested based on Pearson's and Spearman correlation coefficients. All statistical calculations were performed with the R software version 2.14.1 according to the function lm from the R software package [18]. Descriptive statistics was performed in R by deducer package and graphs were visualised by ggplot2 package.

### Evaluation of the procedure on environmental samples

#### Sampling

To evaluate our procedure on actual samples, outdoor sampling was performed at two locations, a wastewater treatment plant (WWTP) which was predicted to have a higher bacterial load [19], [20], and an alpine mountainous area, which was expected to have a lower bacterial load [21].

Impaction holders with sterile 0.7% LMP agarose as an impaction matrix were inserted into portable RCS High Flow air samplers (Biotest, Germany). For analysis based on culture-independent methods, two air samples, designated Air1 and Air2, each of 2 m^3^ were collected in the alpine mountain area (Slovenian Alps, 46°24′7.02″N, 13°36′26.54″E, 760 a.s.l) and nine air samples, each of 2 m^3^ were collected at the WWTP (200,000 population units). In parallel, 0.5 m^3^ of air was sampled in triplicate on the R2A growth media for alpine air and on nutrient agar (NA) for samples taken at three locations in the WWTP to permit characterisation of culturable bacteria from both locations (Table 1).

**Table 1:** Primers and amplification conditions for the detection of *mbt*A or 16S rRNA gene.

Oligonucleotide	Name	Sequence (5′→3′)	Target gene	Amplicon size
Forward primer	U968	AACGCGAAGAACCTTAC	16S rRNA	433 bp
Reverse primer	L1401	CGGTGTGTACAAGACCC	16S rRNA	433 bp
Forward primer	mbtAPH_F	CGACGACGCCCGTGTGATC	*mbtA*	65 bp
Reverse primer	mbtAHA1_R	GCCATCCCGAACACCTGCT	*mbtA*	65 bp

#### Quantification

In samples from the WWTP, quantification of total DNA concentrations and genes was performed by qPCR. Two types of qPCR quantification were performed, a more general method based on 16S rRNA genes and another specific method targeting the *Mycobacterium avium* spp. *hominisuis mtbA* gene - because the WWTP is located next to a pig farm. DNA concentrations were measured with Quanti-It™ dsDNA HS Assay Kit using QubitTM fluorometer (Invitrogen). In the same DNA samples 16S rRNA genes were quantified in triplicate by qPCR (7500 Real-Time PCR System, Applied Biosystems). The 20 µL volume reaction mixtures contained 200 nM primers U968 and L1401 (Table 1, [22]), 10 µL of 2× SYBR Green PCR master mix (Applied Biosystems), 8.6 µL DNAse-free water and 1 µL of genomic DNA. Cycling conditions for real-time PCR were 2 min at 50°C for prevention of DNA carryover, 10 min at 95°C for enzyme activation and initial denaturation, which was followed by 50 cycles of 15 s at 95°C for denaturation and 60 s at 60°C for annealing, extension and data acquisition. A final dissociation step was added to exclude dimer interferences with the quantification. A qPCR standard curve was determined with a series of 10-fold dilutions of pCR2.1 plasmid with inserted partial 16S rRNA gene of *E.coli*, amplified with the same primers. The detection limit was set at a Ct value of 38 that corresponded to 15 copy numbers. 16S rRNA gene copy numbers were calculated from triplicates of up to 500-fold dilutions of DNA samples.

For *mbtA* gene qPCR quantification the 20 µL reaction mixtures contained 600 nM primers mbtAPH_F and mbtAHA1_R (Table 1), 10 µL of 2× SYBR Green PCR master mix (Applied Biosystems), 7.8 µL DNAse-free water and 1 µL of genomic DNA. Cycling conditions for real-time PCR were 2 min at 50°C for DNA carryover prevention, 10 min at 95°C for enzyme activation and initial denaturation, followed by 50 cycles of: 15 s at 95°C for denaturation and 60 s at 61°C for annealing, extension and data acquisition. A final dissociation step was added in order to assess the potential occurrence of dimers. A qPCR standard curve was determined with a series of 10-fold genomic DNA of *Mycobacterium avium* spp. *hominisuis*, amplified with the same primers. The detection limit was set at a Ct value of 36 that corresponded to 6 copy numbers. *mbtA* gene copy numbers were calculated from triplicates of up to 100-fold dilutions of DNA samples.

#### Diversity

For culture-dependent analysis bacteria sampled on R2A solid growth media, taken at the alpine mountain area, were incubated at room temperature for 48 h. Colonies of bacteria were enumerated and pure cultures from all grown colonies were isolated. The culturable bacteria from one of three strips were characterized by sequencing of 16S rRNA genes. The restriction fragment length polymorphism (RFLP) analysis of 16S rRNA genes was used on 33 and 39 colonies from Air1 and Air2, respectively and the 16S rRNA genes from representatives of each RFLP group were sequenced. Shannon-Wiener (*H*) index and rarefaction analysis were calculated using Mothur software with a 97% OTU threshold [23].

For culture-independent analysis, DNA was extracted from agarose according to the established protocol (see final protocol) and a clone library was constructed based on PCR-amplified and -cloned 16S rRNA genes in the pCR 2.1 vector. Accordingly, 25 µL of a PCR mixture consisting of 10× PCR buffer, 31.25 nmol MgCl_2_, 2.5 nmol dNTPs, 1.5 U of Ampli Taq polymerase (Applied Biosystems), 10 pmol of each primer U968 and L1401 (Table 1, [22]), DNAse-free water and 1 µL of genomic DNA were used in PCR amplification. During PCR amplification the following cycling conditions were used: 3 min of denaturation at 92°C, followed by 35 cycles of 30 s at 92°C, 30 s for primer annealing at 54°C, 1 min at 68°C for extension, and a final cycle at 72°C for 7 min on TProfessional temperature cycler (Biometra, Goettingen, Germany). PCR products of 433 bp were separated on the 1% agarose gels, excised and purified through silica columns according to the manufacturer's instructions (Wizard SV Gel and PCR Cleaning System, Promega). Clone libraries were constructed by ligation of purified PCR products in TOPO TA vector pCR2.1 and transformed in electrocompetent *E. coli* Top10 strain (Invitrogen) according to the manufacturer's instructions. From each constructed clone library, 60 clones were randomly selected and 16S rRNA gene inserts in pCR2.1 plasmids were sequenced by using M13f primer (Macrogen, Korea). The closest relatives for given sequences were determined by using the BLAST tool in the GenBank sequences databank and SeqMatch in the RDPII database [24]. The sequences were grouped into operational taxonomic units (OTUs) using a threshold of ≥97% sequence similarity. Shannon-Wiener (*H*) index, rarefaction curves and Libshuff analysis were calculated using Mothur software [23].

### Negative controls

Negative controls on DNA contamination of 0.7% LMP agarose, membrane PES filters and all chemicals, were done simultaneously for bacterial spiking and outdoor sampling. These controls were treated in the same way as samples, total extracted DNA concentrations were measured and 16S rRNA genes detection was performed by PCR and qPCRs.

## Results

### Optimization of DNA extraction from agarose matrices

**Figure 1:**
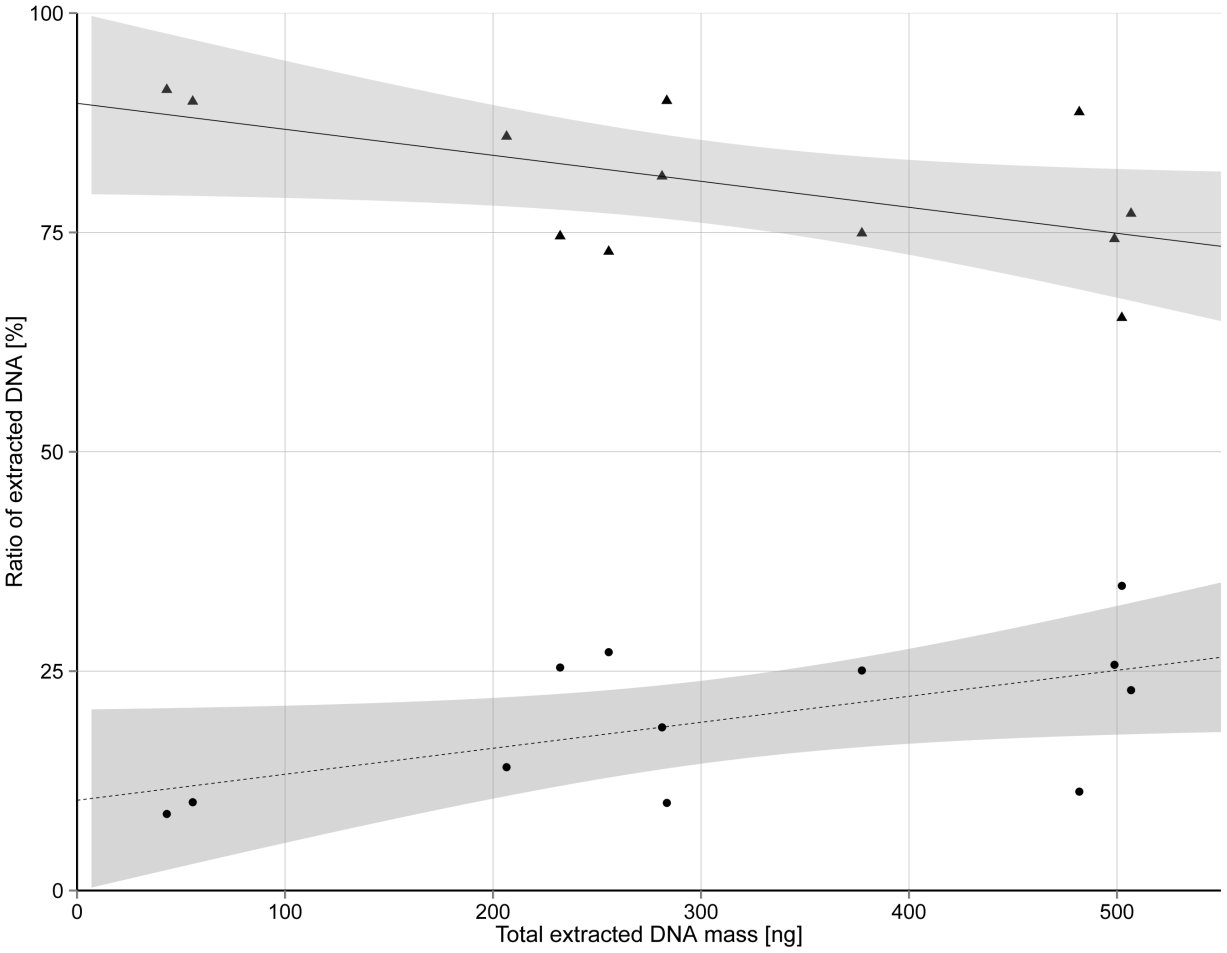
Ratio of total DNA extracted either from retentate or filtrate. Ratio of total DNA extracted either from PES membrane filter (retentate) – (•) or concentrated with ultrafiltration (filtrate) – (▴), after bacterial spiking of agarose followed by enzymatic hydrolysis in relation to the sum of DNA extracted from each individual sample (abbreviated as total extracted DNA mass). The sum of the • and ▴ percentages in each vertical line is 100%, which is represented on the total extracted DNA mass axis. Black lines represent linear trend: filtrate (R^2^ = 0.331, slope coefficient −0.03 and intercept 89.7) and retentate (R^2^ = 0.331, slope coefficient 0.03 and intercept 10.3). 95% confidence intervals for both fitted lines are presented with grey area.

To sufficiently dissolve 8 mL of 0.7% LMP agarose at least 30 min at 65°C or 1.5 min at 100°C was required, as determined by the disappearance of agarose residuals, visible on the membrane filters after the filtration procedure. Acidic and enzymatic hydrolysis both prevented reverse gelling of agarose. However, addition of GuSCN to the acid hydrolysis reaction significantly increased the final sample volume by a factor of at least 2 of the initial sample volume, to approximately 16 mL. Increase in the overall volume after the addition of agarase was ∼110 µL and was deemed to be negligible. Incubation with agarase for 1.5 h showed greater agarose degradation than 1 h incubation, as determined by the higher viscosity in less degraded samples. After both acid and enzymatic hydrolyses, on average 32.5%±11.3% and 19.5%±8.5% (Figure 1) of total DNA mass were isolated from retentates, which showed that most of the cells were lysed during the agarose melting procedure and the released DNA successfully passed through the filter. The DNA in the filtrate had to be extracted, and this was the most conveniently achieved by ultrafiltration. Since the DNA in the filtrate was above 5 kbp, it was determined that ultrafiltration would be at least 50% efficient. In samples treated by enzymatic hydrolysis in which 1.5 min at 100°C was used to dissolve agarose followed by 1.5 h incubation with agarase, the minimum centrifugation time needed to concentrate the sample to a final volume 100 µL, was 20 min. All other samples had centrifugation times between 30 min and 60 min, especially in samples treated with GuSCN. In these cases, the centrifugation time was in excess of 45 min since the overall volume was up to twice that developed in the enzymatic hydrolysis procedure.

### Performance evaluation

#### Yield

**Figure 2:**
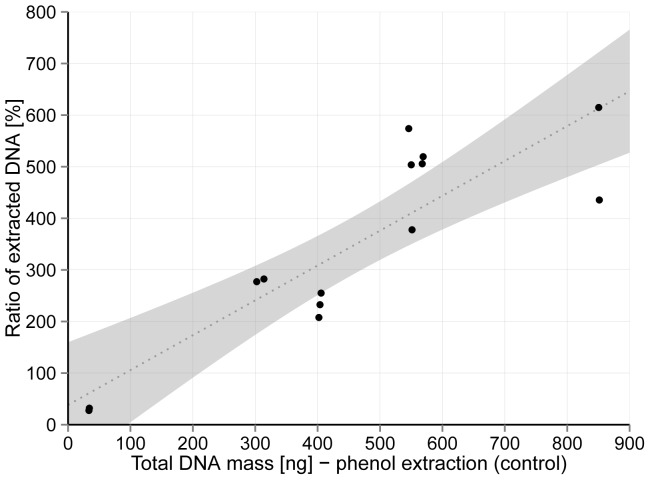
Total DNA mass extracted after bacterial spiking. Linear regression between total DNA mass extracted with developed protocol from spiked agarose matrices and total DNA mass extracted directly from bacterial cells (R2 = 0.76, slope coeficient 0.68, intercept 38.2). 95% confidence interval for fitted line is presented with grey area.

A positive linear regression (R^2^ = 0.76, slope coefficient 0.68, intercept 38.2) was determined between the total DNA mass extracted from spiked agarose matrix and total DNA mass extracted directly from bacterial cells (Figure 2) as well as significant positive correlation based on Pearson's and Spearman coefficient (p<0.0001 for both). Residuals were randomly and normally distributed (Figure S1). Since the coefficient of the regression line is below 1, at higher values of total amount of DNA the extraction yield is lower (Figure 2), while the ratio of DNA mass extracted from retentate was higher (Figure 1). The extraction efficiency was higher than 50% in all tested samples, with an average value of 79.2%±18.0%.

#### PCR amplification efficiency

The PCR amplification efficiency was assessed in spiked as well as actual samples obtained from air with low (alpine region) and high (WWTP area) amounts of cells. The conventional amplification of 16S rRNA genes was successful from spiked samples as well as samples collected outdoors. Approximately 10^3^ CFU/m^3^ were determined at WWTP and 10^1^ CFU/m^3^ at the alpine area (Table 2, Table 3). For efficient amplification, dilutions of at least 10-fold had to be applied to samples taken at the WWTP, whereas in samples obtained from the alpine area or spiked, the PCR amplification could be detected in non-diluted samples.

**Table 2:** Total DNA mass, copy numbers of 16S rRNA gene and *mbtA* gene per m^3^ of sampled air at three locations inside WWTP.

				16S rRNA gene	*Mycobacterium avium* spp. *hominisuis* (*mbtA*)
Sample name	Air sampling location and sampling time	CFU/m^3^	Total DNA mass (ng/m^3^)	Copy numbers/m^3^	Copy numbers/m^3^
AGC-1	Aerated grid chamber – Day 1	-	37.7	2.1E+09±7.3E+08	-
AGC-2	Aerated grid chamber – Day 2	1713±677	33.2	2.1E+09±8.7E+08	215±64
AGC-3	Aerated grid chamber – Day 3	-	26.5	2.3E+08±1.3E+08	-
AB-1	Aeration basin – Day 1	-	14.6	3.8E+07±3.7E+07	-
AB-2	Aeration basin – Day 2	1629±437	36.1	2.1E+08±1.1E+08	337±134
AB-3	Aeration basin – Day 3	-	27.3	6.3E+07±5.7E+07	-
E-1	Entrance to the management building – Day 1	-	12.1	4.2E+07±3.7E+07	-
E-2	Entrance to the management building – Day 2	859±59	24.6	4.3E+07±2.1E+07	884±115
E-3	Entrance to the management building – Day 3	-	26.5	1.7E+07±9.3E+06	-

**Table 3:** CFU per 2^3^ air sample size (CFU±SD) and *H* index determined in clones and isolates from alpine area. Values in brackets are high and low 95% confidence interval.

				*H* index	
Sample name	CFU/2 m^3^	isolates sequenced	clones sequenced	isolates	clones
Air1	89±22	31	50	1.63 (1.90, 1.37)	2.63 (2.26, 2.99)
Air2	96±49	39	56	2.47 (2.75, 2.16)	3.57 (3.80, 3.35)

In all nine samples from the WWTP we extracted enough DNA to measure fluorometrically the total DNA mass which was found to be between 12.1 and 37.7 ng/m^3^ (Table 2). With real time PCR, we detected 10^6^–10^9^ copy numbers of partial 16S rRNA gene per m^3^. Amplification was achieved within a linear range from 50- to 500-fold dilution, with a dynamic range of Ct 22–38, extending from 330 to 1260 pg total DNA/m^3^ at a maximum to 24.2 to 75.5 pg of total DNA/m^3^ at a minimum.

By *M. avium* spp. *hominisuis*-specific qPCR we detected 215 to 628 copy numbers of *mbtA* gene per m^3^ (Table 2). Amplification was achieved within a linear range from 50- to 100-fold dilution, with a dynamic range of Ct 31–36, extending from a maximum of 330 to 1260 pg total DNA/m^3^ to a minimum of 36.1 to 24.6 pg of total DNA/m^3^.

#### Bacterial diversity

**Figure 3:**
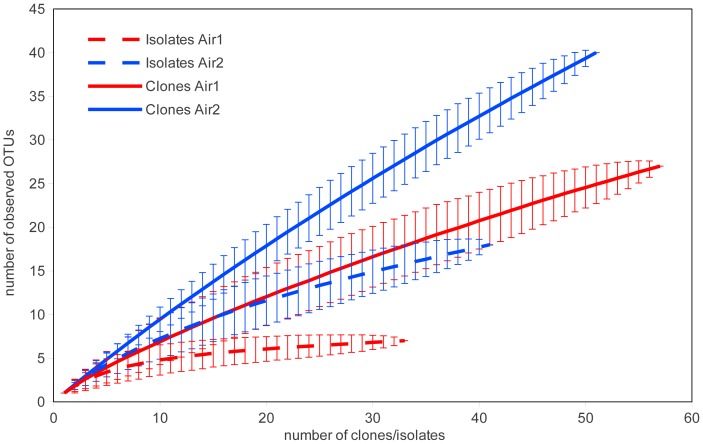
Rarefaction curves from clone libraries and from isolates. Rarefaction analysis of 16S rRNA genes from clone libraries and from isolates, both obtained from alpine air samples. 95% confidence intervals are shown.

The diversity of culturable bacteria was compared to the 16S rRNA gene diversity in the clone library. According to the calculated rarefaction curves and *H* index, the bacterial diversity was higher in both clone libraries than was determined by cultivation (Figure 3, Table 3). The taxonomic assignment of clones and isolates showed sequences in common with the obtained isolates only among *Methylobacterium*, *Acinetobacter* and *Brevundimonas* in Air1 and Air2, respectively (Figure S2, S3, S4, S5). All other clones were found to be exclusive to clone libraries or among isolates. Bacterial diversity of culturable bacteria and clones were significantly different in each location (p<0.05) as determined by Cramer-von Mises test.

### False positives and false negatives

Since air samples contain relatively low quantities of bacterial cells it was necessary to determine the purity of the reagents. After DNA isolation from PES as well as from chemical reagents, the total DNA concentrations were below the detection limit (<0.5 pg/µL of DNA extract).

### Optimized protocol for isolation of DNA from cells sampled from air by impactor on an agarose matrix

**Figure 4:**
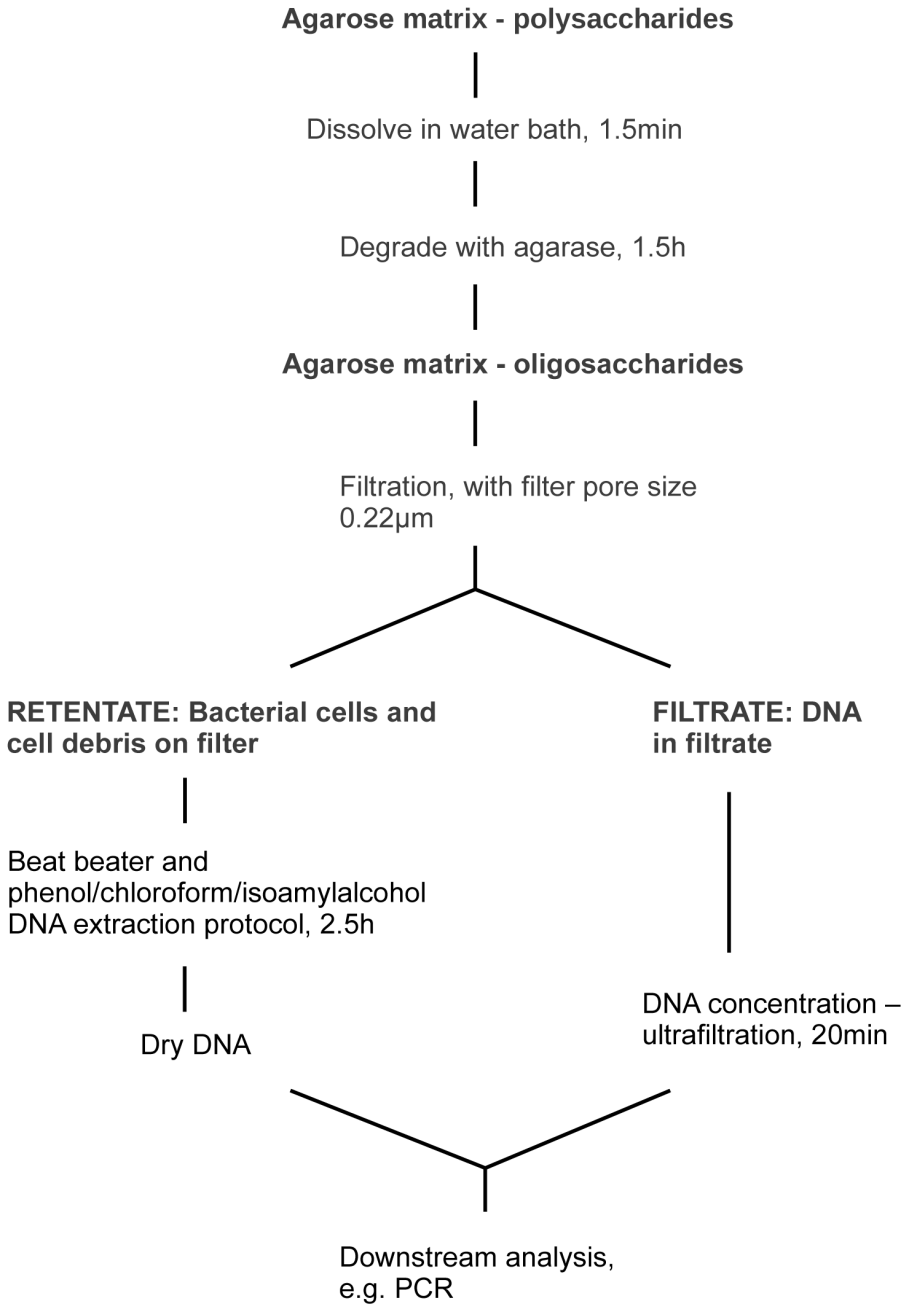
Scheme of the developed protocol.

According to our optimisation of (i) the volume of a melted agarose solution, (ii) sufficient degradation of polysaccharides and (iii) recovery of DNA from filtrate as well as retentate, the final DNA extraction protocol is described here (see Figure 4). LMP agarose (0.7%) from the impactor holder must be completely dissolved in a water bath for 1.5 min at 100°C in DNase- and RNase-free 10 ml tubes (Sarstedt). Dissolved agarose (∼8 mL) is then cooled to 40°C for 10 min and degraded into oligosaccharides by treatment for 1.5 h at 42°C with 7 U of β-agarase (Fermentas - Thermo scientific) per 1 mL of agarose. Degraded agarose was filtered through polyethersulphone (PES) membrane filters with 0.22 µm pore size (25 mm, Milipore) the filtrate being directly collected into ultrafiltration columns (Vivaspin 4, MWCO 10 000, Sartorius). Parallel extraction of DNA from retentate and filtrate is performed to extract DNA from intact bacterial cells and cell debris. DNA from the filter is extracted with phenol/chloroform/isoamyl alcohol according to the protocol of SmartHelix® Complex samples Kit (Sekvenator Ltd., Slovenia). The method combines dissolution of PES filters and lysis of bacterial cells by bead beating (MillMix 20, Tehtnica, Slovenia). The dried DNA pellet isolated from the retentate is resuspended in DNA solution from the filtrate (approx. 100 µL). The filtrate is concentrated beforehand by centrifugation in the ultrafiltration columns at 9000 rpm for approximately 20 min. (Figure 4). Such DNA solutions can be used directly in further downstream analyses.

## Discussion

In clinical microbiology, the Koch postulates restrict the use of methods based merely only on isolation and identification of nucleic acids since disease-causing agents must be isolated and confirmed. However, in some cases molecular methods may be the only feasible choice for investigation of microorganisms which are difficult or impossible to cultivate. Cultivation and molecular methods are not mutually exclusive but rather are complementary. For instance, after molecular detection of highly contagious microorganisms that are hard to isolate, the cultivation methods must be utilized finally to establish the cause of the disease. Methods which utilize both approaches are in such cases highly relevant. Our current understanding of human microbiomes has revealed many previously unknown aspects of microbial interference with the human body and progressively more attention is being paid to environmental microorganisms [8], [25], most of which, unfortunately are currently not culturable. Accordingly, we have developed a method which can be used by both molecular and cultivation-based approaches, and is at the same time highly efficient, does not interfere with downstream applications and can be applied for analysis of quantity and diversity of airborne microorganisms. Since both approaches can be used in parallel this method speeds up the identification of microorganisms and the detection of the source of a particular microorganism by applying fast molecular methods.

The efficiencies of both the bioaerosol collection and nucleic acids extraction, are the most important factors in air investigations, since amount of bacterial cells in air (10^3^ to 10^6^/m^3^) are low in comparison to other environmental samples (in rivers for example, 10^12^ to 10^14^ of bacteria per m^3^ of water is common). Since impactor type air sampler has a predefined efficiencies of aerosol collection [26], [27], only the structure of impaction matrix and DNA extraction efficiency can be optimized. Higher diversity of culturable bacteria was reported after impaction of bioaerosols onto solid nutrient agar than when impacted on solid gelatin as well as when air was impinged into water or filtrated through cellulose acetate or gelatin filters [9]. The developed method that use solid agarose as a support for impaction, which is very similar to nutrient agar support, showed higher DNA extraction efficiencies (79% efficiency, see Figure 2) than it was reported for DNA extraction efficiencies from filters (50% efficiency) [28], currently the preferential method used in culture independent studies. There are no published data concerning DNA extraction efficiencies after direct bacterial spiking in gelatin or mineral oil. However, in the studies in which researchers used these two matrices for bioaerosol sampling and where they compared the estimated number of microorganisms from qPCR to the number of culturable bacteria it resulted in between 1 to 2 [29] and between 2 to 3 [12] orders of magnitude more bacteria determined by molecular approach than they were able to cultivate when collected in gelatin or mineral oil, respectively. If we use the same comparison approach based on our method in which we sampled outdoor air, we determined 3 orders of magnitude more overall bacteria than can be obtained only by cultivation. Comparable results were obtained when samples were collected into liquid by cyclone or glass impingers [9], [30]. Although these results cannot be directly compared to the gelatin and mineral oil based sampling method, one can speculate that agarose based method developed in our study is very efficient in obtaining bacterial DNA, since we determined high ratio between unculturable and culturable bacteria.

Although our method resulted in relatively high DNA extraction efficiencies in spiking experiments, some substantial losses were still observed. Since the same phenol/chloroform/isoamyl alcohol DNA extraction method was used to extract DNA from agarose as well as from bacterial cell suspension, we were able to exclude the influence of the DNA extraction method on the overall DNA loss. We speculate that DNA can be lost (i) during removal of impaction matrix from impaction holders, as has been observed [12] for a mineral oil matrix, (ii) during the ultrafiltration step by passing smaller fragments through filter membrane and (iii) by the attachment of DNA to plastic tubes that were used for agarose melting.

In addition to DNA extraction efficiency, the quality and purity of extracted DNA is important for downstream metagenomic analysis, especially in relation to inhibition of PCR-based amplifications. Such inhibition can be attributed to environmental impurities as well as impurities introduced during the procedure. It is known that agarose can inhibit PCR reactions [7], [31] and therefore, in our procedure the most critical step is the elimination of residual agarose and its degradation products. Agarose must be completely melted so that agarase can access the polysacharide and produce complete hydrolysis after a certain incubation time. Since we did not observe inhibition in spiked samples or in samples taken in the alpine mountain area, the agarose contamination was assumed to be below the PCR interference threshold but in samples collected at the WWTP we observe PCR inhibition which is most likely attributable to inhibitory substances present in the DNA samples [32]. In such cases it is necessary to purify the DNA samples further.

It is known that culturable bacteria represent only a fraction of total bacteria present in air [33] and that by observing only culturable bacteria the actual quantity and diversity of total bacteria will be underestimated. In this regard, the results of our taxonomic assignment of clones obtained from impaction of bioaerosols on agarose matrices show that indeed not all cultured bacteria are reflected in bacteria determined from total extracted DNA. Moreover, the majority of closest representatives of obtained clones belonged to uncultured bacteria. It has to be noted that by molecular methods we observed presence of some pathogens (e.g. *Streptococcus pneumoniae*) or opportunistic pathogens (e.g. *Mycobacterium avium* spp. *hominisuis*) which were absent from culturable bacteria. This can be of extreme importance, since immunosuppressed individuals can be infected, an event which was actually noted by the workers employed at the sampled WWTP and which was severally reported by other researchers (see [34]). Therefore, the method reported here can be of particular interest for monitoring occupational exposures to bioaerosols.

In conclusion, the DNA extraction efficiency of the developed protocol was on average 79.2%±18.0%. We were able to successfully extract enough DNA with good quality to proceed with downstream analysis such as PCR and the method was applied to quantify and to determine diversity of bacteria in air from the WWTP and from air from an alpine environment, which have a high and low bacterial load, respectively. Compared to culturable fractions, culture-independent fractions showed (i) higher number of calculated bacteria, (ii) higher diversity and (iii) large proportion of closest relatives to uncultured bacteria. In future, our DNA extraction protocol should be implemented in air monitoring to link the gap between traditional culture and molecular techniques, which both together deliver highly relevant and complementary information concerning bioaerosols.

## Supporting Information

Figure S1 **Evaluation of the linear regression model for total DNA mass extracted with developed protocol from spiked agarose matrices and total DNA mass extracted directly from bacterial cells.** (A) the residuals errors versus their fitted values, (B) square root of the standardized residuals as a function of the fitted values, (C) Q-Q plot of normal distribution of residuals, (D) standardized residuals as a function of leverage.
(TIF)

Figure S2 **Phylogenetic analysis of partial bacterial 16S rRNA gene sequences of isolates obtained from Air1 sampled at the alpine mountain area.** The tree based on Kimura two-parametric distances was constructed by a neighbour-joining algorithm. *Aquifex pyrophilus* was used as the outgroup. Bootstrap values (1000 replicates) greater than 20% are indicated above the branches. The scale bar represents the 0.05 nucleotide substitution per base.
(TIF)

Figure S3 **Phylogenetic analysis of partial bacterial 16S rRNA gene sequences of clones obtained from Air1 sampled at the alpine mountain area.** The tree was constructed using the neighbour-joining algorithm. *Aquifex pyrophilus* was used as outgroup. Bootstrap values (1000 replicates) greater than 20% are indicated above the branches. The scale bar represents the 0.05 nucleotide substitution per base.
(TIF)

Figure S4 **Phylogenetic analysis of partial bacterial 16S rRNA gene sequences of isolates obtained from Air2 sampled at the alpine mountain area.** The tree was constructed using the neighbour-joining algorithm. *Aquifex pyrophilus* was used as the outgroup. Bootstrap values (1000 replicates) greater than 20% are indicated above the branches. The scale bar represents the 0.05 nucleotide substitution per base.
(TIF)

Figure S5 **Phylogenetic analysis of partial bacterial 16S rRNA gene sequences of clones obtained from Air2 sampled at the alpine mountain area.** The tree was constructed using the neighbour-joining algorithm. *Aquifex pyrophilus* was used as the outgroup. Bootstrap values (1000 replicates) greater than 20% are indicated above the branches. The scale bar represents the 0.05 nucleotide substitution per base.
(TIF)
