# Supplementary figures and images for: The Efficient Method for Simultaneous Monitoring of the Culturable as Well as Nonculturable Airborne Microorganisms
Source: PLoS One. 2013 Dec 20;8(12):e82186. doi: 10.1371/journal.pone.0082186 (PMC3869663; doi:10.1371/journal.pone.0082186)

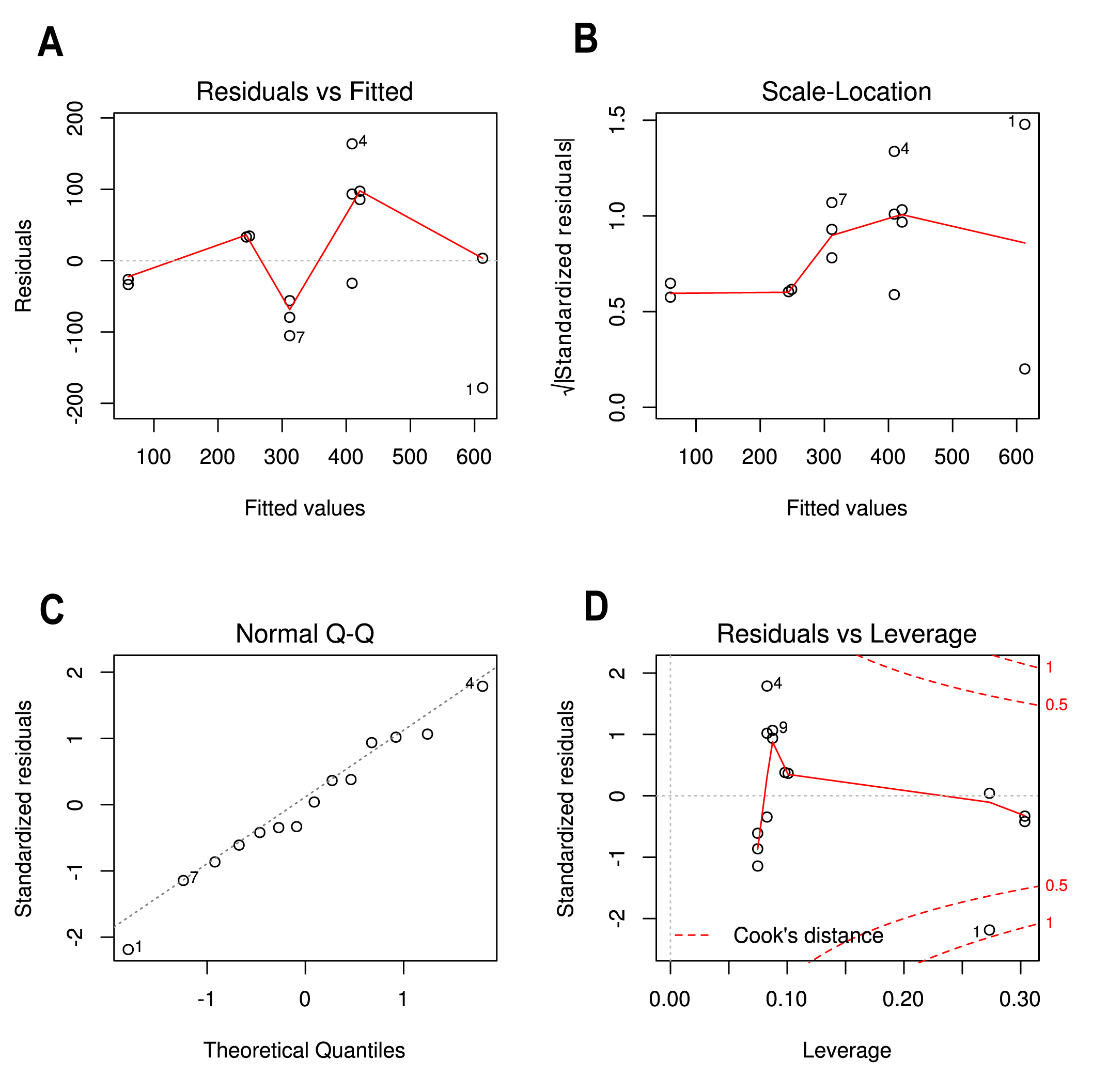

Supplement: Figure S1 — Evaluation of the linear regression model for total DNA mass extracted with developed protocol from spiked agarose matrices and total DNA mass extracted directly from bacterial cells. (A) the residuals errors versus their fitted values, (B) square root of the standardized residuals as a function of the fitted values, (C) Q-Q plot of normal distribution of residuals, (D) standardized residuals as a function of leverage. (TIF) [file pone.0082186.s001.tif]

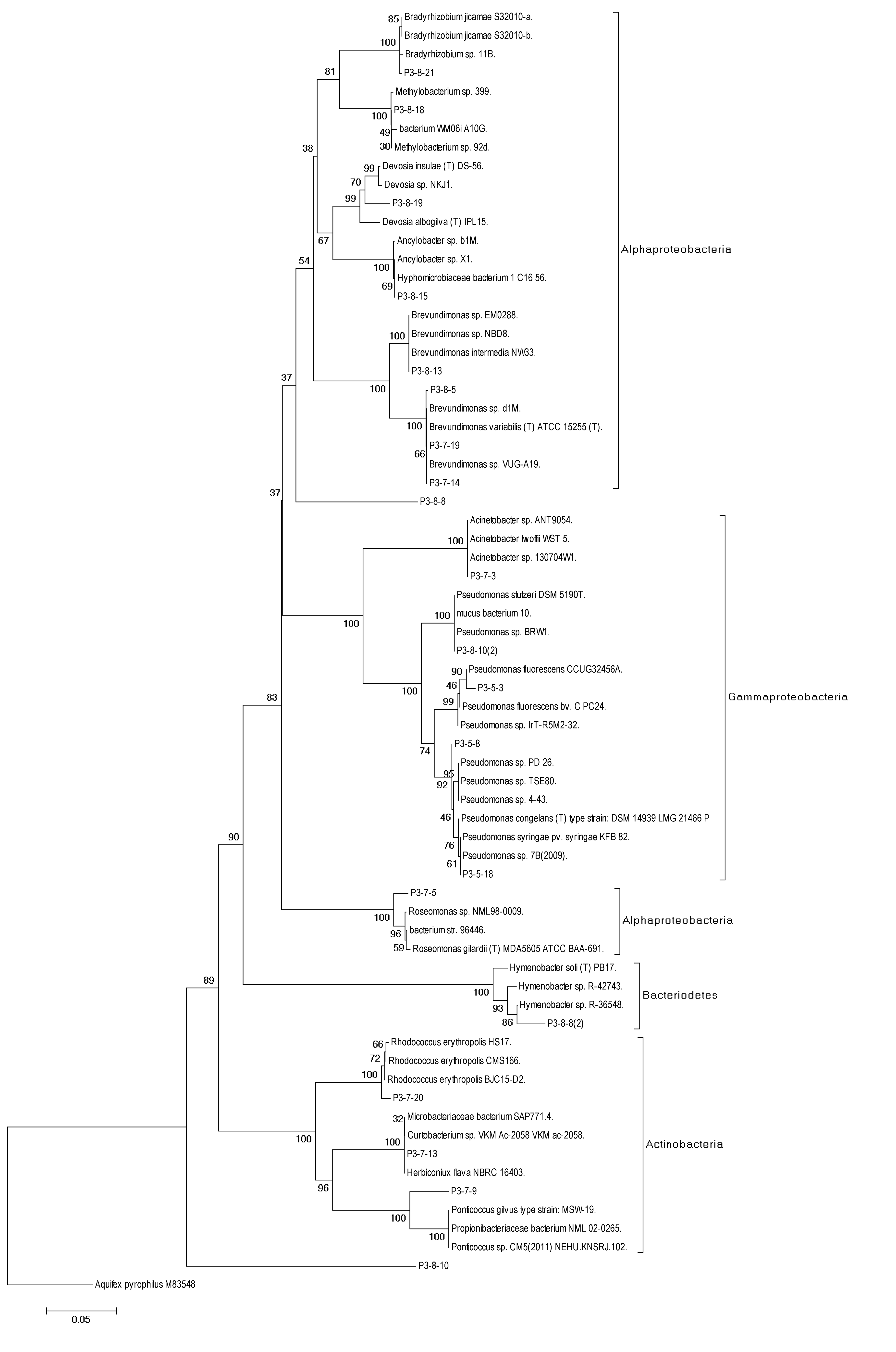

Supplement: Figure S4 — Phylogenetic analysis of partial bacterial 16S rRNA gene sequences of isolates obtained from Air2 sampled at the alpine mountain area. The tree was constructed using the neighbour-joining algorithm. Aquifex pyrophilus was used as the outgroup. Bootstrap values (1000 replicates) greater than 20% are indicated above the branches. The scale bar represents the 0.05 nucleotide substitution per base. (TIF) [file pone.0082186.s004.tif]

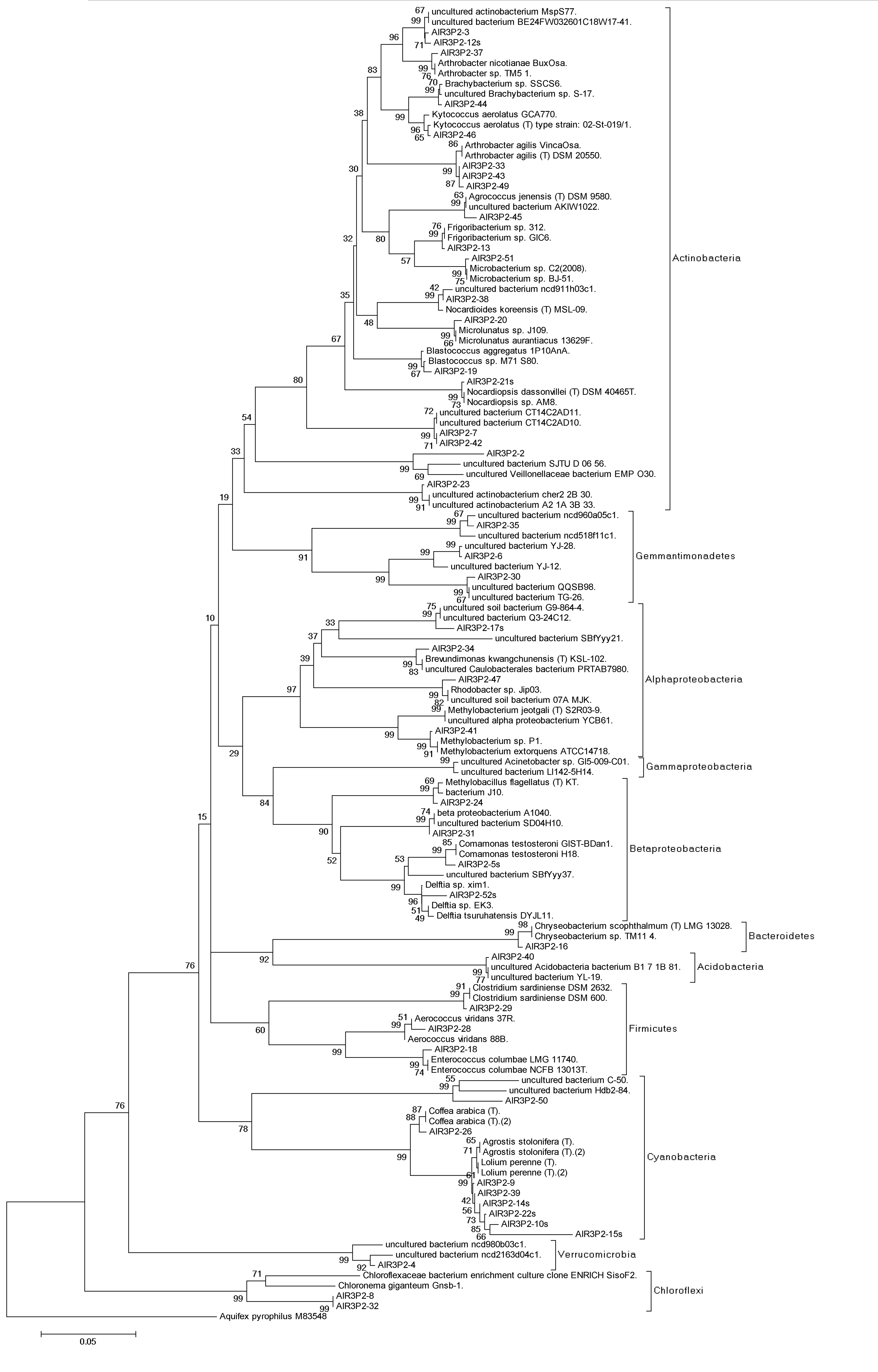

Supplement: Figure S5 — Phylogenetic analysis of partial bacterial 16S rRNA gene sequences of clones obtained from Air2 sampled at the alpine mountain area. The tree was constructed using the neighbour-joining algorithm. Aquifex pyrophilus was used as the outgroup. Bootstrap values (1000 replicates) greater than 20% are indicated above the branches. The scale bar represents the 0.05 nucleotide substitution per base. (TIF) [file pone.0082186.s005.tif]
